# To Evaluate the Damage of Renal Function in CIAKI Rats at 3T: Using ASL and BOLD MRI

**DOI:** 10.1155/2015/593060

**Published:** 2015-03-29

**Authors:** Wen-bo Chen, Long Liang, Bin Zhang, Chun-ling Liu, Hong-jun Liu, Hai-ying Luo, Qiong-xin Zeng, Chang-hong Liang, Guan-shu Liu, Shui-xing Zhang

**Affiliations:** ^1^Department of Radiology, Guangdong Academy of Medical Sciences/Guangdong General Hospital, No. 106 Zhongshan Er Road, Guangzhou, Guangdong Province 510080, China; ^2^Department of Radiology, HuiZhou Municipal Central Hospital, No. 41 E Ling Bei Road, Huizhou, Guangdong Province 516008, China; ^3^Southern Medical University, No. 1838 Dadao Bei Road, Guangzhou, Guangdong Province 510515, China; ^4^F. M. Kirby Research Center for Functional Brain Imaging, Kennedy Krieger Institute, Baltimore, MD, USA; ^5^The Russell H. Morgan Department of Radiology and Radiological Science, Division of MR Research, Johns Hopkins University School of Medicine, Baltimore, MD, USA

## Abstract

*Purpose*. To investigate noninvasive arterial spin-labeling (ASL) and blood oxygen level-dependent imaging (BOLD) sequences for measuring renal hemodynamics and oxygenation in contrast induced acute kidney injury (CIAKI) rat. *Materials and Methods*. Thirteen SD rats were randomly grouped into CIAKI group and control group. Both ASL and BOLD sequences were performed at 24 h preinjection and at intervals of 0.5, 12, 24, 48, 72, and 96 h postinjection to assess renal blood flow (RBF) and relative spin-spin relaxation rate (*R*_2_^*^), respectively. *Results*. For the CIAKI group, the value of RBF in the cortex (CO) and outer medulla (OM) of the kidney was significantly decreased (*P* < 0.05) at 12–48 h and regressed to baseline level (*P* = NS) at 72–96 h. In OM, the value of *R*_2_^*^ was increased at 0.5–48 h (*P* < 0.05) and not statistically significant (*P* = NS) at 72 and 96 h. Conclusions. RBF in OM and CO and oxygen level in OM were decreased postinjection of CM. ASL combining BOLD can further identify the primary cause of the decrease of renal oxygenation in CIAKI. This approach provides means for noninvasive monitoring renal function during the first 4 days of CIAKI in clinical routine work.

## 1. Introduction

Contrast induced acute kidney injury (CIAKI), used to be named as contrast-induced nephropathy (CIN), is a common complication in renal function mostly caused by the administration of contrast media [1, 2]. And it is the most common cause of iatrogenic, inpatient, and drug-induced acute kidney injury (AKI) and the third commonest cause of hospital-acquired renal failure with an incidence of 11% [3–5]. The European Society of Urogenital Radiology (ESUR) Contrast Media Safety Committee proposed the definition of CIAKI as a condition in which a decrease in renal function occurs within 3 days after the intravascular administration of contrast media (CM) without an alternative aetiology.

The cause and mechanism of CIAKI were various, but the three universally acknowledged causes may include kidney arteriolar vasoconstriction resulted from sustained contrast-induced hypoxia in cortex and medulla and the ischemia-mediated oxidative stress causing renal damage [6]. Although many specialists hold that the high-osmolar should be the arch criminal for CIAKI, some studies made a point that low-osmolar and iso-osmolar CM resulted in the same incidence of CIAKI [7, 8].

Nuclear medicine and traditional MR perfusion weighted imaging were universally acknowledged to be useful for measuring kidney perfusion, but both were invasive and required intravenous injection. In recent years, blood flow and tissue oxygen bioavailability, which are founded to be changed in CIAKI as compared to baseline, can be measured noninvasively with MRI techniques called arterial spin labeling (ASL) and blood oxygen level-dependent (BOLD) MRI that does not require using additional markers or tracers [9–14]. Noninvasive ASL and BOLD MR techniques were demonstrated useful to reveal long-term damage in kidney function caused by ionic high-osmolality diatrizoate, particularly in outer medulla [15]. The comprehension of how blood flow and oxygenation change in the early phase has a high clinical relevance for monitoring the transient kidney injury during the course of administration of ionic iodinated contrast agent and evaluating the possibility and extent of induced permanent damage in kidney function. Towards this end, we adapted ASL and BOLD MRI methods for measuring renal hemodynamics and oxygenation in CIAKI rats up to 96 hours after the injection of CM using a 3 Tesla human scanner. Such an approach can be easily expanded to other renal diseases.

## 2. Materials and Methods

### 2.1. Animals

Thirteen male Sprague-Dawley (SD) rats with body weights of 200–250 g were obtained from provincial center for experimental animals. All animals were kept under standard conditions and fed with standard rodent chow and free water.

#### 2.1.1. Ethics

All procedures were not only approved by the local Research Ethics Committee, but also were in accordance with the Guide of the Care and Use of Laboratory Animals published by the US National Institutes of Health (NIH Publication No. 85-23, revised 1996).

#### 2.1.2. Animal Preparation

ALL the rats were injected to receive ionic iodinated contrast agent (Meglumine Diatrizoate, 370 mg/mL, 6 mL/kg) [15] via tail vein injection to induce CIAKI, after six untreated rats of them were scanned as the control. Each rat was fasted for 4 hours before MRI scans. Twenty minutes prior to MRI studies, the rat was anaesthetized with an intraperitoneal injection of 20% urethane (6 mL/kg b.w.). During anesthesia, rectal temperature was monitored to maintain the body temperature at 37 ± 0.5°C using a heating lamp.

### 2.2. MR Studies

All experiments were performed using a 3.0-T whole-body system (Signa EXCITE HD, GE Healthcare, Milwaukee, WI) with a 40 mT/m (200 mT/m-ms) gradient system and a transmit birdcage body coil with 50-mm outer diameter and an eight channel receive-only volumetric rat array (RAPID Biomedical GmbH, Rimpar, Germany), for homogeneous RF transmission and signal detection respectively. The rats were placed head first and supine to make the kidneys in the RF center of the rat array coil. To determine the dynamic change of intrarenal perfusion and oxygenation, the CIAKI rats were scanned 0.5, 12, 24, 48, 72 and 96 h after the injection of CM. At each time point, we got blood samplings from the postcava of the rats to examine renal function.

The details of the pulse sequences used are listed below.

1. *FSE-XL/T1WI Sequence*. Axial Scan Mode = 2D, Grad Mode = zoom, TR = 500 ms, TE = 13.3 ms, Flip Angle = 90°, Bandwidth = 15.6 Hz, NEX = 4.0, FOV = 10.0 × 7.0 cm, Slice Thickness = 2.0 mm, Sap = 0.2 mm, Matrix = 288 × 192, ET = 3, and Scan Time = 94 s.
2. *FSE-XL/T2WI Sequence*. Axial and coronal Scan Mode = 2D, Grad Mode = zoom, TR = 4500 ms, TE = 125.8 ms, Bandwidth = 62.50 Hz, NEX = 6.0, FOV = 10 × 7.5 cm, Slice Thickness = 2.0 mm, Sap = 0.2 mm, Matrix = 288 × 192, ET = 19, and Scan Time = 443 s.
3. *ASL-FAIR-SSFSE Sequence*. Coronal Scan Mode = 2D, Grad Mode = zoom, TR = 2453 ms, TE = 119.8 ms, Bandwidth = 83.3 Hz, FOV = 10.0 × 10.0 cm, Slice Thickness = 2.0 mm, Sap = 0.2 mm, Matrix = 128 × 128, ET = 19, Multislice = 4 slices, Scan Time = 159 s.
4. *BOLD-MFGRE Sequence*: Coronal Scan Mode = 2D, Grad Mode = zoom, TR = 100 ms, TE = 4.0 ms, Bandwidth = 31.3 Hz, FOV = 10.0 × 10.0 cm, Slice Thickness = 2.2 mm, Sap = 0.2 mm, Matrix = 96 × 96, ET = 19, Multislice = 4 slices, Scan Time = 78 s.

### 2.3. Postprocessing

#### 2.3.1. ASL

A four-layer acquisition was performed on both kidneys, with 20 different images at each layer. All images were used to measure renal perfusion by functool-fair software on Advantage Workstation 4.3 GE Medical System (AW4.3_05).

#### 2.3.2. BOLD

Parametric images of *T*_2_^∗^ and *R*_2_^∗^ (equal to 1/*T*_2_^∗^) were obtained on a pixel-by-pixel basis using the Advantage Workstation 4.3 GE Medical System (AW4.3_05). Measurements of *R*_2_^∗^ and *T*_2_^∗^ were obtained including cortex (CO), outer medulla (OM) and inner medulla (IM). The slope of natural logarithm of signal intensity versus echo time equals to relaxation rate *R*_2_^∗^ which is positive correlation with the concentration of deoxyhemoglobin.

**Figure 1:**
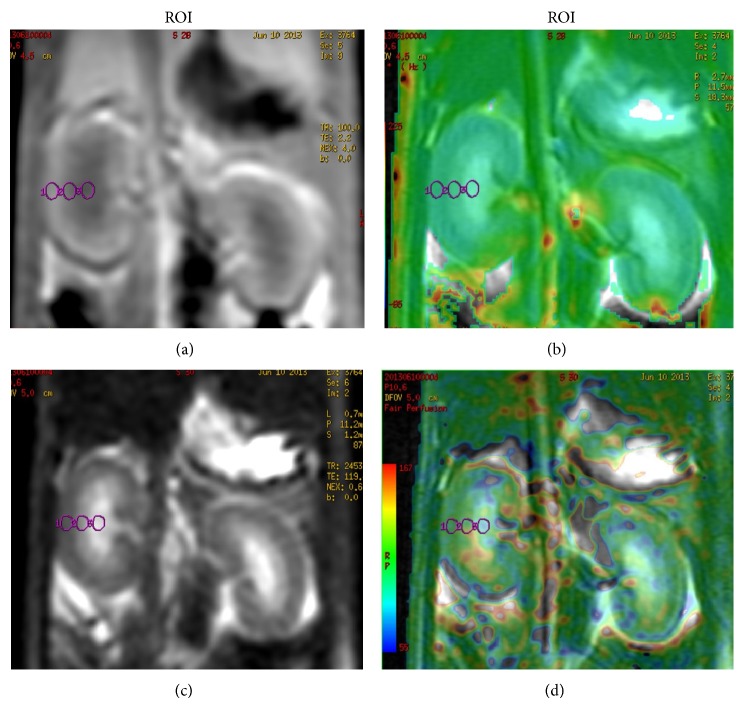
Representative coronal MR image (a) and the *R*_2_^∗^ map (b) acquired using a BOLD sequence. Representative coronal MR image (c) the RBF map (d) acquired using an ASL sequence. The ROI putting on each kidney structure assembles the CO (1), OM (2), and IM (3). In (b) and (d), function parametric maps were overlaid on the top of the corresponding coronal MR image. Software automatically sums the number of voxels, MR signal intensity and calculate the RBF and *R*_2_^∗^ values.

All MR images were evaluated by two radiologists blindly to the group designations. The two radiologists were asked to reach a consensus if there was a discrepancy in the result. The regions of interest (ROIs) were selected at the level of renal hilum of the kidney, with the same size and shape (Figure 1). Each ROI had an area of 3.5–5.5 mm^2^ and contained at least five pixels. ROI was measured in cortex, outer medulla, and inner medulla, without any distortions, artifacts, large vessels, renal collecting system or any incidental renal cysts in the ROIs.

### 2.4. Biochemistry Assessment

The blood collected from the inferior vena cava of rats was centrifuged at the speed of 3500 rmp for 10 min. The obtained serum was measured for the serum creatinine concentration, which reflects the injury of kidney function in the model rats.

### 2.5. Statistical Analysis

Data were analyzed using SPSS 13.0 for Windows. *K* independent samples test and Mann–Whitney *U* test for further comparisons between specific group pairs were used. *P* < 0.05 was used as the criteria for the statistical difference among groups.

## 3. Results

Except for one rat which died at 30 min after intravascular administration of Meglumine Diatrizoate, likely due to overdose of 20% urethane, all other twelve rats completed the entire protocol including the overall ASL imaging and BOLD imaging successfully until 72 h postinjection Besides, there were anther 3 rats died before 96 h time point MRI scan likely due to fasting and narcotized over and over again at each time point and resulted in a poor condition and even died. All the data were successfully recorded. Both the RBF values and *R*_2_^∗^ values could be calculated from each time point map.

**Figure 2:**
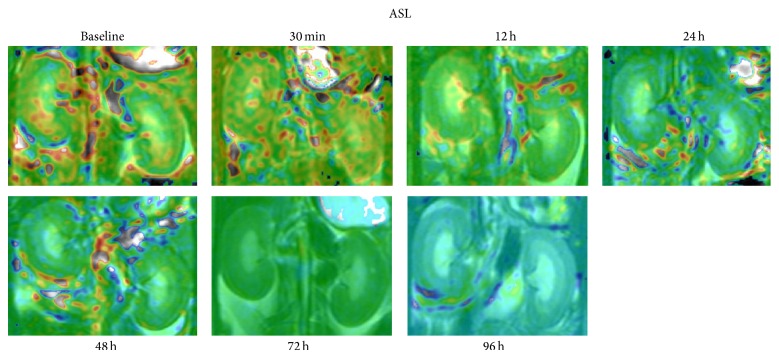
Time course of RBF changes in the kidney of a representative CIAKI rat over a 96 h period after injection of iodinated CM using an ASL sequence. Representative coronal SSFSE MR maps include CO, OM, and IM of each kidney before and 0.5, 12, 24, 48, 72 and 96 h after infusion.

**Figure 3:**
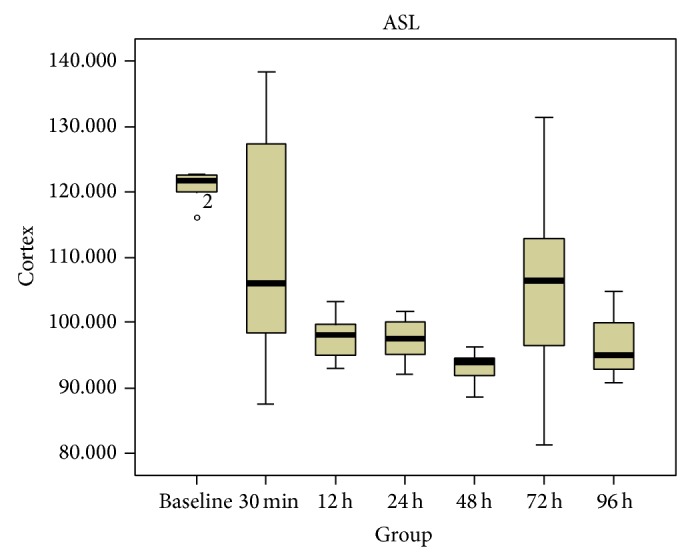
Box plots of RBF values in CO. The top and bottom of the boxes indicate the first and third quartiles, respectively. The length of box represents the interquartile range, within which 50% of the values were located. The solid line within each box is the median. The error bars show the minimum and maximum values (range). The circle mean the outlier. This box plot implied that the 50% of the values at 12 h, 24 h, 48 h were lower than baseline.

**Figure 4:**
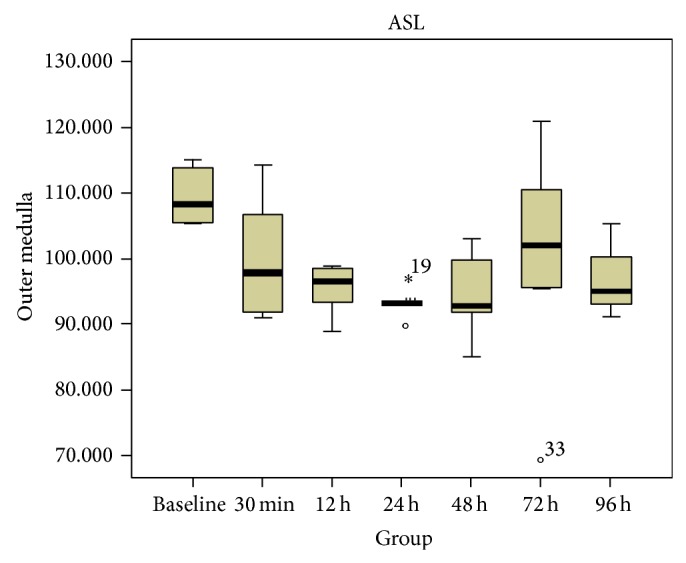
Box plots of RBF values in OM. The circle and asterisk mean the outlier and extremum respectively. This box plot implied that the 50% of the values at 12 h, 24 h, 48 h were lower than baseline.

The spatial resolution (i.e., 0.78 × 0.78 mm) of all the ASL maps used for RBF measurement was good enough to distinguish among the CO, OM, and IM of the kidney (Figure 2). The RBF values in the CO, OM and IM in the rats before and after the injection of iodinated contrast agent were shown in Table 1. In the CO and OM of the kidney, the values of RBF at 12, 24, and 48 h after injection were found significantly decreased (*P* < 0.05 versus baseline, *n* = 6, resp.; Figures 3 and 4), while those at 72 and 96 h gradually regressed to a level close to baseline (*P* = NS versus baseline).

**Figure 5:**
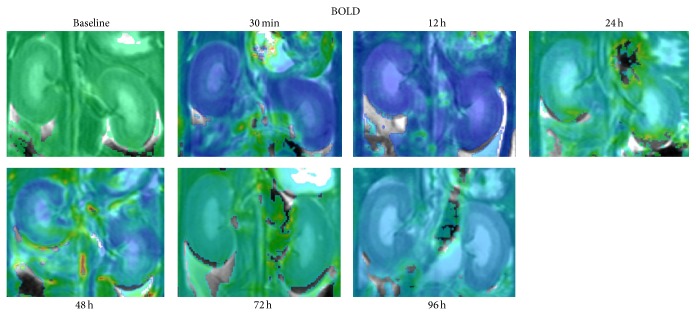
Time course studies in BOLD sequence of baseline and CIAKI rats over a 96 h period after injection of iodinated CM. Representative coronal MFGRE MR maps include CO, OM, and IM of each kidney before and at 0.5, 24, 48, 72 and 96 h after CM injection.

**Figure 6:**
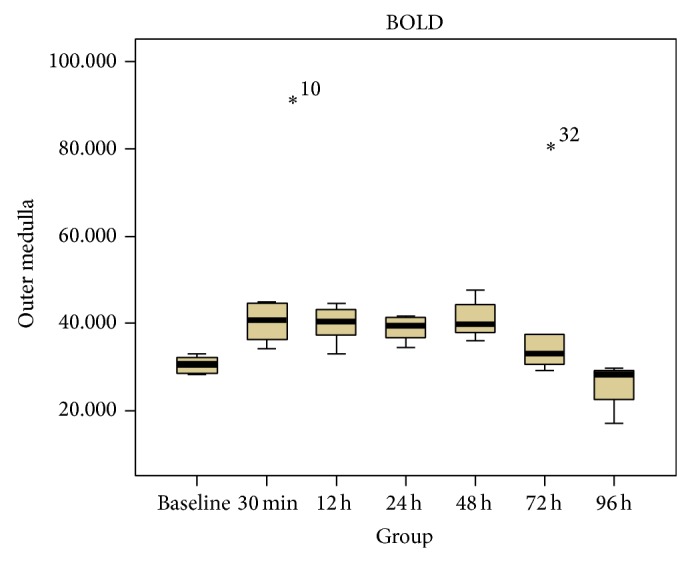
Box plots of *R*_2_^∗^ values in OM. The asterisk means the extremum. This box plot demonstrate that the 50% of the values at 0.5, 24 and 48 h were larger than baseline, while at 72 h and 96 h time point the overlap was present with baseline.

All the BOLD images also had enough spatial resolution (i.e., 1 × 1 mm) to distinguish CO, OM and IM of the kidneys (Figure 5).  Table 2 shows the average change of oxygen level in the regions of CO, OM and IM in the CIAKI rats. In the kidney OM, the value of *R*_2_^∗^ were greatly increased between 30 min to 48 h (*P* < 0.05 versus baseline, *n* = 6, Figure 6), but fell back to baseline after 72 h (*P* = NS versus baseline). The CM did not produce statistically significant changes in *R*_2_^∗^ within the kidney CO and IM (*P* = NS versus baseline).

The value of RBF and *R*_2_^∗^ were also assessed using the exact same procedure in six untreated rats before CM injection. In addition, there was no statistical significance in the mean RBF and *R*_2_^∗^ during the time course of our study, proving that the changes we found in the CIAKI rats using ASL and BOLD methods were the direct consequences of kidney injury.

**Figure 7:**
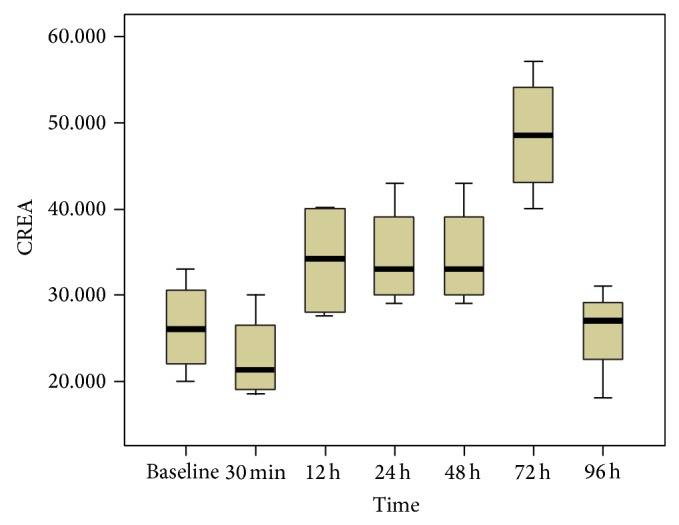
The concentration of serum creatinine before and 0.5, 12, 24, 48, 72 and 96 h after injection of CM. The concentration of serum creatinine was significantly increased only at 72 h (*P* < 0.05 versus baseline), whereas at other time points the differences did not reach statistical significance (*P* = NS versus baseline).

**Figure 8:**
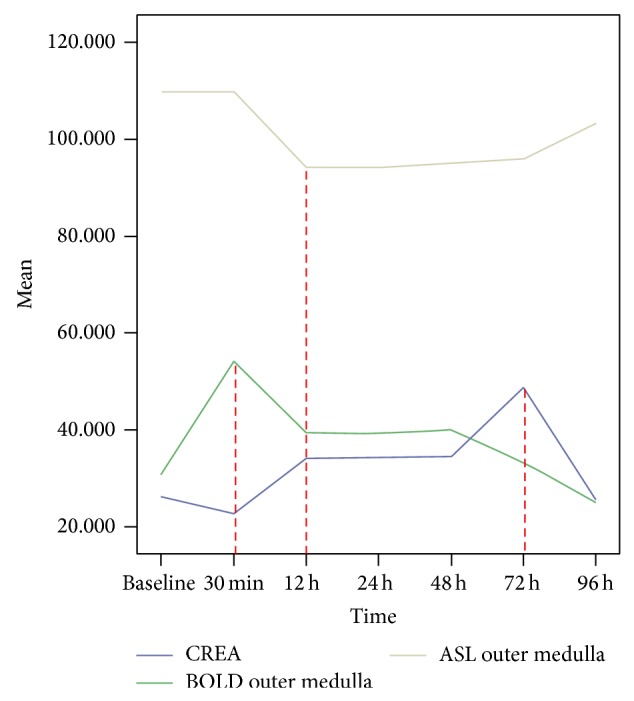
The *R*_2_^∗^ value, RBF value of OM and the concentration of serum creatinine. The superior line demonstrated that the RBF value was significantly decreased at the 12 h time point and last to 48 h (*P* < 0.05 versus baseline). The value of middle line demonstrated *R*_2_^∗^ were greatly increased at 30 min and last to 48 h (*P* < 0.05 versus baseline). The inferior line demonstrated the concentration of serum creatinine was significantly increased only at 72 h (*P* < 0.05 versus baseline). The changes of serum creatinine values were relatively later than both the changes of *R*_2_^∗^ value and RBF value.

To further verify whether kidney was injury after injection of iodinated CM and allows correlation with disease severity, blood serum creatinine was measured. The serum creatinine concentration reached the maximum at 72 h (*P* < 0.05 versus baseline, Figure 7), whereas at other time points the difference did not reach statistical significance (*P* = NS versus baseline). As shown in Figure 8, the changes of serum creatinine value were relatively later than both the changes in *R*_2_^∗^ value and RBF value.

## 4. Discussion

CIAKI was defined as the decrease in renal function after CM injection, using SCr as a marker. However, SCr is a surrogate marker for glomerular filtration and does not provide the complete information of kidney function. For example, it was reported that when the impaired nephrons is less than 50%, no change or only marginal change of SCr could be observed [2]. Therefore a comprehensive method that describes the whole spectrum of kidney function in CIAKI is highly desirable. As previous study [15] reported that ASL and BOLD MRI were useful to reveal renal damage caused by ionic high-osmolality diatrizoate, our study tried to use this two MRI techniques to monitor the dynamic change of renal function in CIAKI model.

In order to measure the kidney function more accurately, we have made several improvements of MR scan. In particular, we implemented a pulse sequence to measurement both pre- and postinjection CM. And we also utilized a free breathing protocol acquisition. In addition, we chose a coronal orientation to avoid major feeding vessels. With these improvements, we measured the time-evolution of RBF and *R*_2_^∗^ value in both intact and CIAKI kidneys over a broad range of function.

Our results demonstrated a significant decrease of RBF value in both CO and OM, which is similar to previous reports [16]. But it should be noted that the RBF value of CO in intact animals obtained in our study are slightly lower than those reported by Liu et al. [16], (i.e., baseline level our RBF value was 120.84 ± 2.34 mL/100 g/min versus 124.89 ± 6.60 mL/100 g/min; 24 h group our RBF value was 97.35 ± 3.51 mL/100 g/min versus 123.49 ± 9.16 mL/100 g/min). This may partly be due to the variation in acquisition and processing.

Some studies about CIAKI focused on the acute effects of iodinated CM on renal hemodynamic distribution (i.e., from the first hour after CM injection to 24 h) [15, 17, 18]. Our study revealed a prolonged decrease of RBF value in both CO and OM last to 48 h after the injection of diatrizoate, and gradually returned to a level close to baseline at later time (72 and 96 h post-injection). As we know, signal intensity on FAIR-ASL is attributed to blood flow including both artery and vein. Therefore, a persistent stasis-state of venous blood cells after iodinated CM injection may probably increase the cardiac afterload and decrease the blood speed and renal perfusion, which must have an important contribution in the decease of RBF value [19]. And acute vasoconstriction in the renal capillaries caused by the accumulation of the injected iodinated agents may be the deeper reason. Besides, the mechanism why RBF value decreased, especially in the outer medulla, could be explained as follows. Firstly, CM could directly decrease blood flow of renal medullary by increasing plasma viscosity. Secondly, CM with high concentration could cause a pronounced osmotic diuresis that distends the tubules and collecting ducts, leading to renal swelling, increasing intrarenal venous pressure [20] and decreasing outer medullary blood flow. Taking account of persistent stasis-state of venous blood cells into consideration, it can be explained why the RBF value of OM was the lowest among the kidney structures measured.

Originating from the *T*_2_^∗^ effect of oxyhemoglobin/deoxyhemoglobin, the *R*_2_^∗^ in tissues measured by BOLD negatively correlates with the tissue content of oxygen (PO_2_) [21]. On CIAKI rats, from 30 min to 48 h, our data demonstrated a significant increase in *R*_2_^∗^ only in OM of kidney using BOLD MRI, indicating a decrease of oxygen level, which was agreed with other studies [22]. Several mechanisms can be attributed to this result. First, as diatrizoate is a hyperosmotic CM, the osmotic effect of agent could elevate the amount of sodium collected at the thick ascending limb of the loop of Henle, which in turn accelerate the active transportation of cations from the loop of Henle [23] and cause an increase of oxygen utilization. Second, due to the high viscosity of the circulating CM, the aggregation of red blood cells may occur, resulting in a decrease of oxygen delivery indirectly by slowing intrarenal blood flow [24, 25]. Finally, iodinated CM could induce medullary vasoconstriction, directly resulting in renal hypoxia [26, 27]. Besides, *R*_2_^∗^ can also be influenced by many pathological conditions, such as iron concentration, the oxygenation ability of hemoglobin, or hematocrit. But in our study, we measured the *R*_2_^∗^ value at different postinjection times as compared to that preinjection from the same rat, in which iron concentration, the oxygenation ability of hemoglobin, or hematocrit is likely to be on the same level. By this way, we wanted to minimize the influence of alternative causes to the *R*_2_^∗^.

What is more, the purpose of why we chose ASL combined with BOLD in monitoring renal damage was to help further identify whether the decrease of renal oxygenation was accounted for either the decrease of blood perfusion or the increase of oxygen consumption. In our study, the increase of *R*_2_^∗^ were greatly increased in 30 min after CM injection in OM, while the RBF was no difference in this time. Therefore we may identify in rough that the increase of oxygen consumption played an more important role in the decrease of renal oxygenation.

In patients, SCr level commonly reaches the peak within 2–5 days after CM administration [28]. Our result demonstrated that the peak of SCr occurs at approximate 72 hours (3 days) after the CM injection, indicating the time course of kidney injury in our animal model is well correlated with patients. Our study implies that both the BOLD and ASL sequence might find the renal injury began no later than 12 h, which was much earlier than blood serum creatinine changes. So, we might conclude that both ASL and BOLD techniques were much more sensitive than SCr in detecting the renal injury after CM injection.

On untreated animals, variation of both RBF and *R*_2_^∗^ values, either among the different kidney structures or over time in the same structure, were small, indicating ASL and BOLD techniques used in our study are reliable for monitoring the damage caused by CM injection, as well as other kidney diseases. As these two MR techniques have been much more widely used in some clinical regimes, our approach may be quickly translated to the patient study for monitoring the damage of renal function in a variety of kidney diseases, especially in CIAKI that we focus on here.

As discussed above we might conclude that ASL combining BOLD could provide much more convictive datas for revealing the primary cause of the decrease of renal oxygen (PO_2_) in CIAKI. It would certainly help clinicians to give the right medicines to their CIAKI patients, by telling them which is more important between oxygen consumption and blood perfusion decrease.

## 5. Clinical Application

Evaluation of the damage of iodinated CM on kidney hemodynamics and oxygenation is critical for better comprehension and prevention of renal hypoxia diseases. BOLD and ASL MRI provide a rapid and accurate means for evaluation of kidney function of most of the common kidney diseases, such as CIAKI, diabetic nephropathy, and kidney transplant. In addition, these techniques can also be used to monitor the therapeutic responses of treatments to common renal hypoxia disease.

## 6. Our Study Has Some Limitations

Our study has several limitations, which may need future investigation. While we measured the serum creatinine level to validate our MRI findings, a histological analysis of renal morphology may be useful to identify the regions of injury. However, because both RBF and *R*_2_^∗^ change back to normal after 96 hours, the histological analysis after the final imaging time point may not be useful. Besides, our study did not take the effect of iodinated CM on the tissue *T*_1_ relaxation into consideration because it took such a long time to measure kidney *T*_1_ relaxation with an IR sequence. In the future study, we will continue to work on improving the accuracy of the measurement and the quality of images by refining our imaging protocol.

In conclusion, we have successfully employed noninvasive ASL and BOLD MRI to acquire high-quality images using 3.0T MRI clinical scanner, allowing the measurement of renal hemodynamics and oxygenation. Our results revealed a significant and prolonged decrease of RBF in OM and CO and also a decreased oxygen level in OM after CM injection, indicating that ASL and BOLD MRI are potentially useful in the clinic for noninvasive assessing of renal function in the case of CIAKI.

## Figures and Tables

**Table 1:** RBF value of pre- and postinjection of CM (mL·100 g^−1^·min^−1^).

Group	Cortex	Outer-medulla	Inner-medulla
Baseline (*n* = 6)	120.84 ± 2.34	109.26 ± 4.17	111.36 ± 5.22
30 min (*n* = 6)	110.62 ± 18.78	99.84 ± 8.95	107.36 ± 18.13
12 h (*n* = 6)	97.89 ± 3.69^∗^	95.37 ± 3.74^∗^	109.32 ± 20.41
24 h (*n* = 6)	97.35 ± 3.51^∗^	93.62 ± 2.20^∗^	108.56 ± 11.85
48 h (*n* = 6)	93.19 ± 2.64^∗^	94.11 ± 6.31^∗^	107.36 ± 8.39
72 h (*n* = 6)	105.83 ± 16.79	100.10 ± 17.29	91.66 ± 20.88
96 h (*n* = 3)	106.15 ± 16.1	103.41 ± 11.51	104.17 ± 5.55

*K* independent samples test, respectively, and Mann–Whitney *U* test for further comparisons between specific group pairs. ^∗^ *P* < 0.05.

**Table 2:** *R*_2_^∗^ value of pre- and postinjection of CM (mean ± SD; Hz).

Group	Cortex	Outer-medulla	Inner-medulla
Baseline (*n* = 6)	29.09 ± 3.22	26.12 ± 1.92	26.03 ± 4.27
30 min (*n* = 6)	36.58 ± 7.09	39.37 ± 4.57^∗^	36.36 ± 9.84
12 h (*n* = 6)	33.99 ± 4.27	39.86 ± 4.37^∗^	38.57 ± 13.01
24 h (*n* = 6)	35.90 ± 7.61	38.85 ± 2.75^∗^	29.54 ± 5.68
48 h (*n* = 6)	36.59 ± 8.34	40.94 ± 4.23^∗^	36.29 ± 7.12
72 h (*n* = 6)	29.48 ± 2.30	32.39 ± 2.89	30.03 ± 4.70
96 h (*n* = 3)	31.22 ± 2.67	24.98 ± 6.99	22.91 ± 9.06

*K* independent samples test, respectively, and Mann–Whitney *U* test for further comparisons between specific group pairs. ^∗^ *P* < 0.05.
